# Musculoskeletal Changes Across the Lifespan: Nutrition and the Life-Course Approach to Prevention

**DOI:** 10.3389/fmed.2021.697954

**Published:** 2021-08-31

**Authors:** Domenico Azzolino, Giulia Carla Immacolata Spolidoro, Edoardo Saporiti, Costanza Luchetti, Carlo Agostoni, Matteo Cesari

**Affiliations:** ^1^Department of Clinical and Community Sciences, University of Milan, Milan, Italy; ^2^Geriatric Unit, IRCCS Istituti Clinici Scientifici Maugeri, Milan, Italy; ^3^Specialization School in Geriatrics, University of Milan, Milan, Italy; ^4^Pediatric Intermediate Care Unit, Fondazione IRCCS Ca' Granda Ospedale Maggiore Policlinico, Milan, Italy

**Keywords:** aging, sarcopenia, bone, obesity, frailty, inflammation, early life, exercise

## Abstract

Aging is characterized by the progressive decline of muscle mass and function, the so-called sarcopenia. Also bone loss is widespread among older people. Sarcopenia and osteopenia/osteoporosis are associated with several adverse outcomes including falls, risk of fractures, functional decline, frailty, and mortality. Recently, the life-course approach to prevent or delay functional decline has become very popular. Regarding musculoskeletal health, there is suggestive evidence that acting during critical or sensitive periods of life in which each person build-up its biological reserves may influence the rate of functional decline in the later stages of life. A life-course approach to musculoskeletal health should take place during early life when plasticity allows more easily the attainment of the peak of the musculoskeletal system driven by environmental stimuli. The rate of the subsequent decline will depend on the peak previously reached. Nutrition and physical exercise are important environmental factors that can influence musculoskeletal development by favoring and maintaining peak bone and muscle mass and strength. Here we provide an overview of body composition changes occurring across the lifespan and strategies based on nutrition and physical exercise to support musculoskeletal health as well as minimizing losses during older life.

## Highlights

– Geriatrics and Pediatrics are commonly seen in antithesis as they occupies the two extremes of life
– During early life each person rapidly accumulates his/her functional capacities in body functions or structures to reach a peak or a plateau at maturity
– Maintaining musculoskeletal health and preventing early losses is pivotal during adult life
– In older life, minimizing losses is crucial
– Implementing a holistic approach, based on nutrition and physical exercise, that people can apply during the life course may optimize the functional ability during aging.

## Introduction

People are living longer but only a few years are still lived without disability (1). The traditional models of care, which were built and remain centered on single disease treatment, are unprepared to manage the complexity of older individuals characterized by chronic comorbidities and mutually interacting syndromes (2, 3). Indeed, there is a need for a more comprehensive and appropriate assessment of the aging population. Advancing age is accompanied by a progressive decline in many functions which cannot be explained by chronological age per se. Sarcopenia, defined as the progressive loss of muscle mass and strength (4), is one of the most serious health concerns in older people. However, sarcopenia can also occur earlier in life in combination with a variety of health conditions. Furthermore, genetic and environmental factors acting during the life-course may influence the decline of muscle mass and strength commonly seen with aging (5).

Phenotypic changes occur very quickly during the first years of life, then stabilize during young adulthood, and accelerate once again with aging (6). During early life, each person accumulates his/her biological reserves which influence the degree of functional ability during older adulthood (7). In the last decade, the life-course approach to prevent or delay functional decline has become very popular (1). The life course approach encompasses both biological and environmental factors acting during gestation, childhood, adolescence and adulthood and their influence in health status, functions and diseases during older life (8). Implementing a holistic approach to prevention that people can apply during their lifetime may optimize functional ability trajectory during aging (9). Recently, the World Health Organization (WHO) introduced the concept of intrinsic capacity which is defined as the composite of all physical and mental capacities that an individual can draw upon the lifetime (10). This new construct may potentially change the current conduction of clinical practice, shifting from a disease-centered toward a function-centered approach (11). In the context of intrinsic capacity, vitality represents the biological background of every individual encompassing complex and dynamic biologic systems which sustain life and functioning. The capacity of any individual expressed by cognitive, locomotor, sensory, psychological domains, is the phenotypical and functional manifestation of this biological background. Based on this concept, designing trajectories of capacity could ideally allow intercepting early influences on late life, and, consequently, implement a personalized plan of intervention (12).

Nutrition, an important contributor to the vitality domain, is a key determinant of health in all age groups, beginning from pregnancy and early childhood and extending throughout the lifespan (6). The quality of the diet over the life-course has been closely related to the incidence of sarcopenia (13). Indeed, (early) nutritional interventions may be able to reduce the incidence of sarcopenia or revert it, potentially improving the individual's intrinsic capacity (11, 13). In other words, it has been repeatedly proposed that inadequate early nutrition may lead to the impaired development of repair systems, suggesting that rates of aging may be determined at the very earliest stages of the life (14).

Geriatric and pediatric specialties occupy the two extremes of life, without formal connections (15). However, both pediatricians and geriatricians look at the person's health in a multidimensional way. As early as 1914, Ignatz Nascher defined senility as a “second childhood”. Nascher stated that no function, organ, or tissue looks exactly within these two periods of life. Indeed, aging is not a regressive process but a progressive one. However, older people show frequently reliance on others (especially those who are frail or institutionalized) as well as pediatric ones. This review article is intended to close the gaps between the two specialties by providing an overview of changes in body composition occurring during the lifetime with a special focus on specific nutrition and physical activity intervention strategies throughout the lifetime, aimed at preventing and delaying the functional decline in musculoskeletal system seen with the aging process.

## Musculoskeletal Changes

Both muscle and bone are highly malleable tissues responding to the environment during the life-course. The two tissues that develop during adolescence, reach a peak in density around the third decade of life, which is maintained in midlife and then declines with aging (16, 17). With aging, there is a progressive decline in muscle mass, strength, and functionality, the so-called “sarcopenia”. In fact, after the fourth decade of life, there is a progressive decline in muscle mass (i.e., 1–2% per year) and strength (i.e., 1.5% per year) (18). However, it has been suggested that muscle mass and strength in older people does not reflect only the rate of loss but also the peak reached during early life (19). In particular, adolescence represents a window of opportunity for musculoskeletal health since this period is characterized by profound changes in body composition resulting in the rapid accretion of both bone and muscle mass. These changes are largely driven by hormonal factors and differ between genders. Indeed, in males the highest levels of testosterone and IGF-1 determine a largest increase in both muscle mass and strength compared to females (20). Despite the timing of pubertal events varies widely among individuals, in females the largest increase in fat-free mass is observed nearly at the age of 15, while in men between 12 and 15 years of age. In both genders, a rapid increase in total body fat is seen but in males is less marked given the concomitant fastest accretion in fat-free mass (21).

There are several mechanisms that concur to the development of sarcopenia. These include malnutrition, physical inactivity, hormonal changes, inflammation, increased catabolism, and anabolic resistance, myocyte's loss, reduced satellite cell number and function, loss of α-motor neurons, mitochondrial dysfunction, and insulin resistance (22, 23). Particularly, insulin resistance through reducing the ability to use the available proteins may result in metabolic alterations associated with type 2 diabetes further exacerbated by sarcopenia (23–25). Interestingly, it has been reported a positive association between birth weight and both muscle mass and strength, which is even maintained during the lifespan (26, 27). Additionally, low birth weight has been consistently associated with type 2 diabetes later in life (28). Indeed, these findings could provide an additional explanation for the association between low birth weight and the incidence of sarcopenia during aging probably through the mediation of insulin resistance. Also genetic and other early life factors (i.e., early growth, longer duration of breastfeeding) have been associated with muscle mass and strength (29). Other than birth weight, it has been reported that also prepubertal and pubertal growth may influence both muscle strength and physical performance later in life (i.e., midlife) (30, 31).

In recent years, above all, inflammation and mitochondrial dysfunction received particular attention as a major determinant of sarcopenia (32, 33). The detrimental effects of persistent inflammation and mitochondrial damage are seen in a variety of pathological conditions characterized by metabolic alterations including diabetes, insulin resistance, and cardiovascular diseases (32). The accumulation of mitochondrial damage and a chronic inflammatory state along with oxidative stress during the lifespan may be the precursors of a variety of age-related metabolic diseases. Indeed, inflammatory status could be regarded as a function of an individual, an early determinant of the aging process which is impacted by diet (6).

On the other hand, bone mass steadily increases during childhood, then rapidly accelerates during adolescence to reach a peak at around 20 years of age (34, 35). However, given the increased levels of estrogens, adolescent females experience a more rapid increase in bone mass than males (20). After the age of 60, a progressive decline in bone mineral density (BMD) of nearly 1–1.5%/year is seen (36). By the age of 70, bone mass is reduced by nearly 30–40% (37, 38). In midlife women, the most important risk factor for bone loss is menopause, since after that the normal bone turnover cycle is impaired by estrogen deficiency, which explains the more pronounced bone loss in the female gender compared to males (20). In particular, across the lifespan women experience a loss of about 50 and 30% of trabecular and cortical bone respectively. Nearly half of the overall bone loss in women is experienced during the first 10 years after menopause (38, 39). According to the WHO criteria, bone loss is defined by the so-called “T-score”, a standardized measure that compares BMD to the average values of young healthy women. Indeed, osteopenia is defined by a T-score between −1 and −2.5 while osteoporosis by a T-score ≤ -2.5 (40). Osteopenia and osteoporosis are also too prevalent conditions during aging (16). Not surprisingly, osteoporosis and sarcopenia frequently occur simultaneously, even leading to the creation of a so-called “osteosarcopenia” condition. Like every other age-related condition, osteoporosis and sarcopenia show a common background in the biology of aging (16). To date, physical inactivity, as well as nutritional deficiencies, may lead to a decline of both tissues (36). Additionally, inflammatory states, endocrine alterations, fat infiltration, metabolic derangements, vitamin D deficiency, comorbidities, and genetic factors are all involved in the pathogenesis of both conditions (i.e., muscle and bone loss) (41). Interestingly, it has been reported that genetic traits determining the peak of bone and muscle mass during early life, may influence the trajectory of both tissues late in life. Across the lifespan, hormonal dysfunctions (i.e., low levels of testosterone in men and estrogen in women) are also known to negatively affect both muscle and bone (16, 42).

## Adipose Tissue Changes

**Figure 1:**
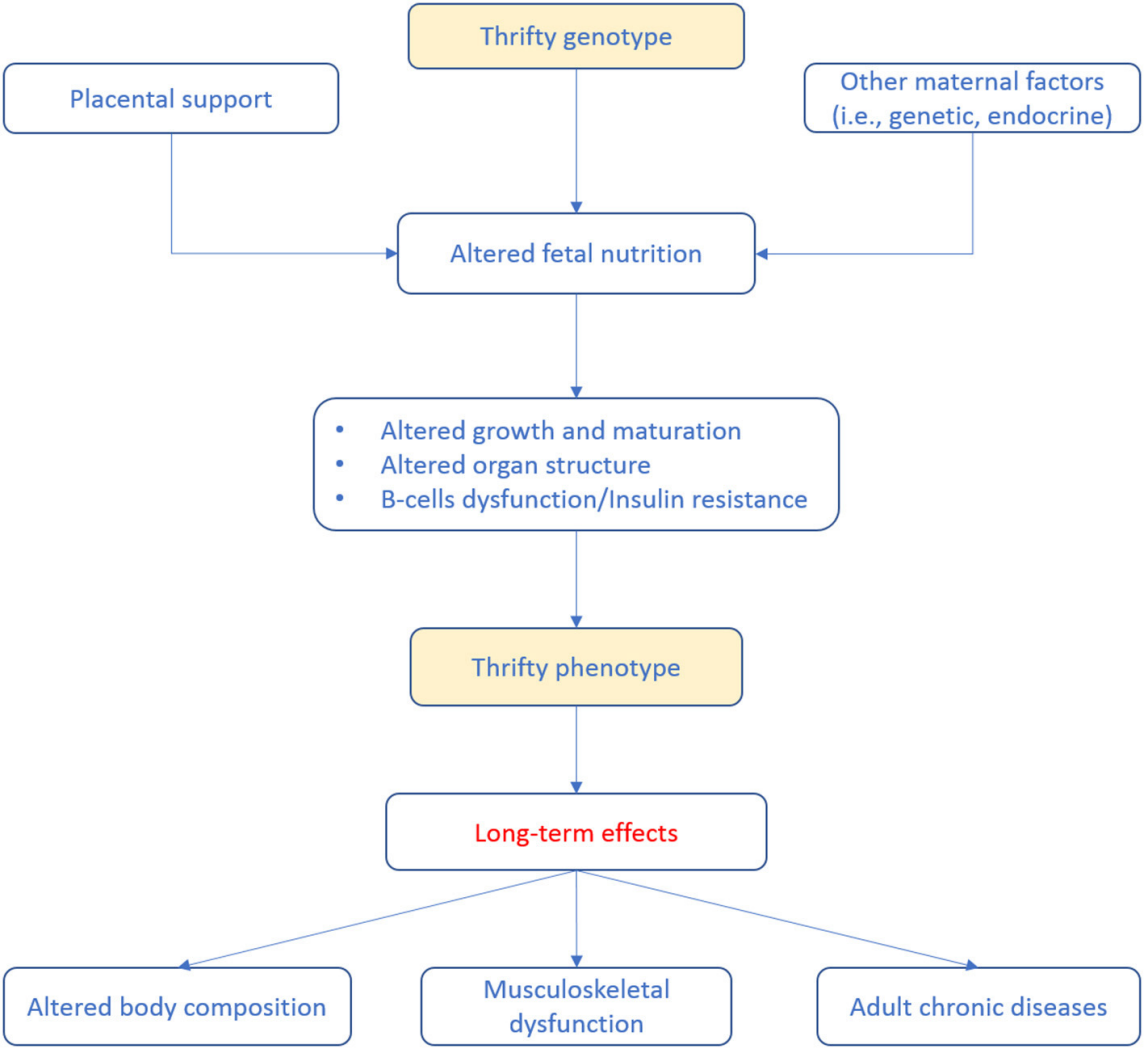
Life-course events predisposing to adult diseases according to the so-called thrifty phenotype hypothesis (53). Poor infant growth, as a consequence of in utero undernutrition, may lead to alterations in glucose-insulin metabolism that, coupled to the effects of obesity, aging and physical inactivity, promotes the development of adult diseases.

Adipose tissue is a fundamental component of body composition and not only an inert body fat storage. In fact, adipose tissue is today recognized as an active endocrine organ mediating several metabolic processes and secreting a variety of adipokines and cytokines regulating systemic inflammation (43). As a consequence, adipose tissue abnormalities may have long-term negative effects on musculoskeletal health probably through the mediation of inflammatory processes, metabolic dysregulation, and altered insulin sensitivity (44–47). Alterations of adipose tissue composition can occur as early as during fetal life and can persist during adulthood (47). Indeed, there is evidence that high birth weight, the so-called fetal macrosomia, is associated with the development of alterations in body composition (i.e., obesity) during adulthood potentially contributing to muscle decline (47, 48). During infancy and early childhood, particular attention should be paid to the growth acceleration. Regarding adipose tissue, it is important to monitor the BMI growth curve which typically is described as *U*-shaped. Infant's BMI tends to reach a peak at around 6–9 months of age and then progressively decreases until 5–7 years of age, from then the BMI curve gradually increases once again delineating the so-called “adiposity rebound” (49, 50). Over time, several studies reported an association between an earlier adiposity rebound and an increased risk of being obese during adulthood as well as a close relationship with non-communicable diseases (49, 51, 52). The complex relationship between body composition alterations during early life and the long-term health effects can be explained by the so-called “thrifty phenotype hypothesis” (Figure 1).

Accordingly, poor nutrition during early life may results in a relatively down-expression of the functional units within splanchnic organs (in particular, pancreas and liver). Therefore, it has been associated with endocrine and metabolic adaptations of the fetus to survive (e.g., low vs. high glucose and energy supply). These adaptations, matched with a higher than programmed energy intake and unfavorable lifestyles in the later phase of life, may further accentuate negative processes, including fat accumulation in organs (i.e., liver) and lean tissue (i.e., muscles). Altogether, these phenomena lead to a consequent decrease in insulin sensitivity and glucose tolerance and reduced muscle mass (46, 54, 55). Adiposity trajectories of z scores (weight-for-height and BMI) have been significantly associated with higher fasting insulin and homeostasis model assessment of insulin resistance (i.e., HOMA-IR). In particular, higher insulin resistance at 14 years of age have been reported in those subjects in which adiposity remained high (56). Lawlor et al. (57) reported that low birth weight, low offspring birth weight, short leg length, high adult BMI, and greater adult waist-to-hip ratio were all independently associated with adult insulin resistance. On contrary, another study has not found associations between birth weight and HOMA-IR, while reported that adult lifestyle and body composition were associated with larger variances in insulin secretion and HOMA-IR (58). It has been widely reported that overweight and obese children are more likely to have these conditions during adulthood, with early development of chronic diseases (i.e., type 2 diabetes and cardiovascular diseases) (59–61) and a negative impact on musculoskeletal health (47).

Regarding older life, concomitantly to muscle mass decline, there is also a progressive increase in adipose tissue. Body fat distribution changes too, with an increase in visceral abdominal fat compared to the subcutaneous abdominal fat (62). Additionally, aging is also associated with fat infiltration of the muscle and bone marrow inducing apoptosis of the myocytes and osteocytes (36). Intramuscular fat infiltration, given its lipotoxic effects, can exert detrimental effects on muscle strength and quality also affecting mobility function (63, 64). Chronic low-grade systemic inflammation, which represents a hallmark of aging, has been indicated as one of the main factors responsible for muscle decline in older people (65). Indeed, the abnormal secretion of these inflammatory mediators by the adipose tissue may further exacerbate the muscle decline in those individuals with obesity. Interestingly, it has been suggested that pro-inflammatory processes occurring during the life-course may determine the inflammatory trajectory later in life (66), thus potentially influencing musculoskeletal health in older life. Therefore, obesity and chronic inflammation should be managed with earlier interventions aimed at targeting early modifiable factors given their negative effects on musculoskeletal health later in life.

## Interventions

**Figure 2:**
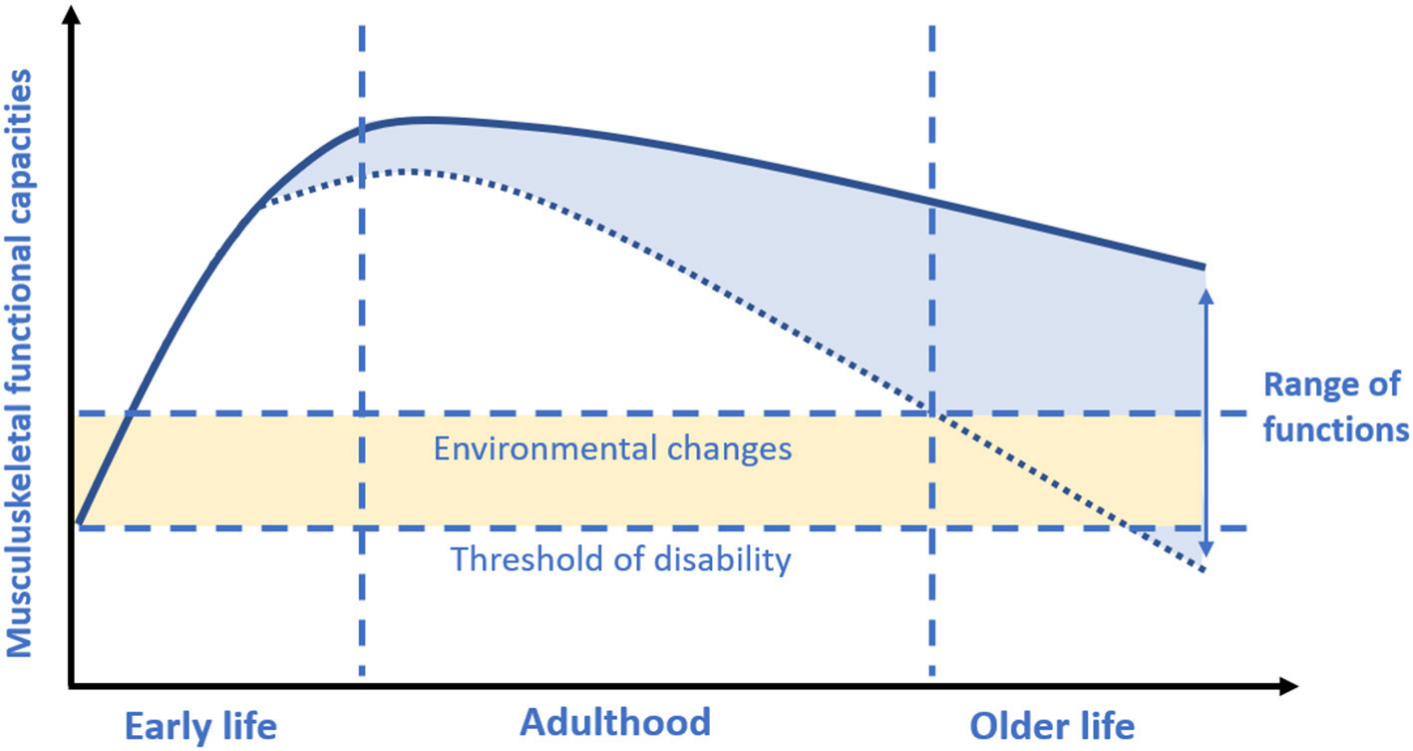
Musculoskeletal functional capacities across the lifespan. Based on concepts and findings by Kuh (7), Sayer et al. (19), and Cruz-Jentoft et al. (17). During early life each person rapidly accumulates his/her functional capacities in body functions (i.e., muscle) or structures (i.e., bone mass) to reach a peak or a plateau at maturity (i.e., nearly at the end of adolescence period). Afterwards, gradually starts a decline with age. The greater the peak, the slower the consequent decline. However, it should be considered that the rate of decline may be also largely driven by environmental factors during adult life.

There is suggestive evidence that acting during critical or sensitive periods of life in which each person build-up its biological reserves may influence the rate of functional decline in the later stages of life. The life-course approach to musculoskeletal health should begin during early life when plasticity allows more easily the attainment of the peak of the musculoskeletal system driven by environmental stimuli. However, some lifestyle factors during adulthood also play an active role in maintaining musculoskeletal health and minimizing musculoskeletal decline. Indeed, the rate of the subsequent decline will depend on the peak previously reached, but also on strategies promoting muscle and bone health later in life (67) (Figure 2).

Among various interventions that can be implemented, nutrition and physical exercise seem to be the most promising (68). The promotion of physical exercise and adequate intake of certain nutrients during physical development may maximize the chances to achieve a higher peak of musculoskeletal mass and strength, controlling the rise of fat within muscle fibers, with a consequent less pronounced decline later in life. During childhood and adolescence, healthy habits are more easily acquired than at later ages. However, if, on the one hand, it is crucial maximizing the peak of muscle mass and strength during early life, maintaining the muscle and bone in adult life and minimizing losses during aging is also pivotal.

### Nutritional Interventions

Nutrition is a key determinant of health in all age groups. Growing evidence suggests that environmental stimuli, such as diet, particularly during pregnancy and early life, and later in life, can act as a determinant of health-related outcomes during aging (69). Both under and over nutrition must be prevented, and particular attention should be paid to micronutrient deficiencies. It is important to note that nutritional interventions can occur at two different levels, with either a preventive or therapeutical approach. The preventive strategy is directed to anticipate possible macro- or micronutrient deficiencies during a defined moment in life. Other than this, therapeutical strategies are aimed to correct a manifested deficit.

#### Pregnancy and Early Life

Early nutritional interventions aimed at setting the basis for a lifelong healthy life can start before conception by favoring the achievement of healthy weight in women. Nutritional intervention during pregnancy should focus on favoring balanced nutrition and food rich in critical nutrients. It is interesting to note that during pregnancy, micronutrient requirements increase more than energy requirements as they mediate important developmental functions for the fetus (70). The WHO recommends the supplementation of both iron (30–60 mg/day) and folic acid (400 mcg/day) during pregnancy for the prevention of maternal anemia, preterm birth, low birth weight, and sepsis (71). Folate supplementation is also recommended before conceiving to promote neurodevelopment (72). Regarding iron supplementation, it has been pointed out that daily supplementation may be limited by the lack of compliance and safety concerns in those with an adequate intake. For this reason, intermittent regimens for iron supplementation have been proposed as a more acceptable strategy (73). Other nutritional recommendations include eating ocean fish twice a week or supplementation with long-chain ω-3 polyunsaturated fatty acids, such as DHA (300 mg/day), as it may help reduce the risk of preterm birth (70). The supplementation of other micronutrients is not recommended, nonetheless, it is important to strictly monitor and correct eventual deficiencies before the deficit becomes clinically evident. For instance, vitamin B12 deficit may negatively impact neurodevelopment and fetal growth and should not be overlooked. The role of vitamin D has also been the object of many studies. It represents the principal mediator of maternal calcium homeostasis and therefore influences the bone development of the fetus. Vitamin D deficiency during pregnancy was linked to osteopenia in newborns and reduced bone density in childhood. Therefore, particular attention should be paid to vitamin D status during pregnancy (74, 75). After birth, it is important to promote a balanced diet and the loss of excessive weight gain in women. Nutrition in infants and young children should aim to achieve the correct weight gain according to the age growth standard. Over time, several studies reported a difference in body composition among breastfed (BF) infants and formula-fed (FF) infants. Gale et al. (76), in a systematic review and meta-analysis, showed that fat-free mass in FF infants was higher in the first year of life while fat mass was lower at 3 and 6 months compared to BF infants. On contrary, at 12 months they found a higher fat mass in FF children. Recently, Rodríguez-Cano et al. (77) documented that exclusive or predominant breastfeeding resulted in an increased fat mass at 6 months compared to those who were not exclusively BF. However, a longer duration of breastfeeding has been associated with subcutaneous fat and not with visceral fat distribution (78, 79). It could be assumed that the higher fat mass observed in BF infants during the first months of life may represent a protective factor for the subsequent weaning period (76). The higher fat mass in BF infants may thus reflect an optimal phenotype resulting in the protection from obesity occurrence in late life (80, 81). Furthermore, a more rapid weight gain has been observed in FF infants compared to BF infants at 3 and 6 months of age in both genders and between 6 and 9 months in girls only (82). Growth acceleration/rapid weight gain is to be prevented as it is associated with lifetime risk. Victora et al. (83) reported an association between rapid growth and greater fat mass at 18 years of age, regardless of the age in which rapid growth occurs. The WHO recommends exclusive breastfeeding for 4–6 months as the preferred method of infant feeding. Interestingly, Robinson et al. (84) reported an association between greater exposure to breastfeeding and higher grip strength in older life. If lactation is not possible for the mother, infant formula with a low content of protein is to be preferred as formula with high protein content are linked to a (mild) higher risk of rapid weight gain and risk of overweight (85). Regular animal milk is not recommended for the first year of life given its high amount of proteins. As for complementary foods, it should be initiated not before 17 weeks and not later than 26 weeks of age (70). A greater variety of foods should be offered immediately to infants for the health advantages of diet diversity in the medium and long term (86). Finally, in the first year of life and early childhood, the intake of simple sugars and salt should be limited (87).

During childhood and later in adolescence nutritional interventions should promote balanced nutrition and the quality of diet. Both children and adolescents are considered at risk of malnutrition given the increased energy and nutrients demand of the body for its development. Indeed, an adequate amount of energy and nutrients is required to sustain the growth spurt (88, 89). Particular attention should be paid to proteins, calcium, and vitamin D, which are essential to maximizing both peak bone mass and muscle mass and strength peak (90). Vitamin D deficiency is widespread among children and adolescents (91). For this reason, in many developed countries and especially in those in which sunshine exposure is poor, vitamin D is added to many food products (e.g., milk, breakfast cereal, flour) (92). Ward et al. (93) and Foo et al. (94) found an association between low vitamin D levels and lower grip strength and muscle power in adolescents. However, the results of randomized controlled trials in which vitamin D was supplemented in adolescents are controversial. El-Hajj Fuleihan et al. (95) reported a significant increase in lean mass after vitamin D supplementation in premenarcheal girls, while no significant changes in grip strength were found. On contrary, vitamin D supplementation in postmenarcheal girls and adolescent boys has not proven effective in increasing lean mass and muscle strength (95, 96). Dietary proteins beyond muscle accretion play important functions in bone health during childhood and adolescence. Proteins are a source of amino acids, which are important in the formation of the bone matrix and intervene in the stimulation of IGF-1 that promotes bone formation (90). However, excess protein intake has been associated with childhood obesity and therefore it should be strictly monitored (97). According to the US Institute of Medicine, the recommended intake for female and male adolescents should be 1,300 mg/day for calcium, 600 IU/day for vitamin D, and is 0.85 g/kg body weight/day for protein (98). Also, iron requirements are increased during adolescence to support the high amount of muscle and higher hemoglobin levels in both genders, as well as to replace menstrual losses in females (89). However, the indications in females of reproductive age are difficult due to the wide distribution of women with higher menstrual losses.

Overweight and obesity are widespread conditions among children and adolescents who are more prone to being overweight and obese as adults (99). During the adolescence period, there is a change of eating behaviors through a net shift toward convenience foods that are rich in saturated fatty acids and sodium and less consumption of healthy food (i.e., fruits, vegetables, dairy whole grains) (89). Such unhealthy behavior is associated with the risk of developing overweight and obesity. Indeed, given the deleterious effects of obesity (i.e., inflammation and early development of chronic diseases) which negatively affects also muscle health and are likely to persist into adulthood, weight management is pivotal during this delicate period. Primary care-based interventions, if properly addressed, can positively contribute to the prevention of unhealthy habits as well as obesity in children and adolescents. Also, school-based interventions may be very useful since usually a high amount of time is spent in the school environment (100).

#### Adult Life

The continuity of a life-course approach can be pursued also during adult life since this period is characterized by the major occurrence of chronic diseases. The adult phase of life can be seen as a critical period in which both preventive strategies and treatment of manifested pathologies can be implemented. In other words, addressing adult risk factors (i.e., under- and over-nutrition, physical inactivity) may be a complementary strategy in the prevention and treatment of chronic conditions reducing both morbidity and mortality (101). Evidence for the role of adult nutrition on the musculoskeletal decline at older ages is still limited. Sabia et al. (102) found an association between unhealthy behaviors during midlife (i.e., low consumption of fruit and vegetables, physical inactivity, smoking, and unmoderated alcohol consumption) and slower gait speed 17 years later. Stenholm et al. (103) documented that excess body weight in midlife is a predictor of muscle strength decline in old age (i.e., after 22 years of follow-up). They also reported an association between marked weight loss and accelerated decline in grip strength. Of note, adherence to the Mediterranean diet has been associated with a slower decline in physical function (104, 105).

#### Older Life

The implementation of early preventive strategies is of particular interest. However, nutritional targets need to be constantly pursued during older life in order to preserve muscle and bone and to delay the functional decline. It is well recognized that older people need more protein to counteract muscle decline than young and adult individuals, mainly because of a declined anabolic response and increased catabolism (23). It is widely acknowledged that the traditional recommended dietary allowance for protein intake (i.e., 0.8 g/kg body weight/day) for all adults is not adequate for older people (23, 106). Indeed, it is recommended a protein intake of at least 1.0 g/kg body weight/day to maintain muscle mass in older people. In presence of acute or chronic illnesses, it is recommended that the protein intake should be increased up to 1.2–1.5 g/kg of body weight/day, while in presence of highly catabolic conditions it may be increased up to 2.0 g/kg of body weight/day (23, 106). Regarding protein source, animal-based proteins are suggested to induce a higher anabolic response than those plant-based proteins, because their higher content of leucine and a greater digestibility (22, 107). Whey proteins (i.e., fast digested proteins) seem to greater stimulate muscle protein accretion than casein (slow digested protein) and soy proteins (23). It is also pivotal an adequate amount of energy since if caloric intake is not sufficient, body fat and muscle are catabolized to provide energy (13). For what concerns caloric provision, it is therefore recommended a guiding value for energy intake of 30 kcal/kg of body weight/day (108). What is more, both the amount of energy and proteins should be adjusted according to the individual's nutritional status, physical activity level, clinical conditions, and preferences (108). It has been also suggested that a high amount of protein per meal (i.e., 25–30 g per meal containing at least 2.5 g of leucine) is required for anabolic response in older individuals (23). Vitamin D deficiency is very common in older individuals and it has been associated with reduced muscle mass and strength; the correction of deficiencies should thus be actively pursued (109, 110). Furthermore, supplementation of Vitamin D and calcium (i.e., at least 1,000 IU/day of vitamin D and 1,000–1,200 mg/day of calcium) is generally recommended in case of deficiency to preserve bone mass and to prevent osteoporotic fractures (111, 112). Protein intake plays a key role also for bone health through the life-course. Physiologically, there is a steady turnover and remodeling of the bone protein matrix which account for nearly a half of bone volume and one third of its mass. Indeed, an adequate amount of proteins is required to support both the formation and maintenance of bone mass (113). A recent systematic review (114) suggested that a protein intake higher than the current RDA may help in reducing the risk of hip fracture as well as may promote BMD maintenance in older people. Finally, in recent years calorie restriction has received growing interest. In particular, calorie restriction without malnutrition seems to have strong anti-inflammatory properties (115), reduced oxidative stress, health span improvement and lifespan extension (116). However further (especially human) studies are needed to elucidate the mechanisms and efficacy as well safety and feasibility of calorie restriction.

### Physical Exercise Interventions

The promotion of habitual physical activity is essential from early life, as it benefits musculoskeletal tissue development and helps to maintain a healthy body weight throughout life. What is more, during adolescence physical activity has a significant influence on the growth of the fat-free mass, with early prevention of fat accumulation within muscles that unfavorably affect the glucose/insulin axis and homeostasis. Several hormonal factors like testosterone, growth hormone, and IGF-1, which are stimulated by physical activity, in turn, promote muscle mass accretion during adolescence (117). Recently, Hao et al. (118) reported an association between moderate and vigorous physical activity with greater skeletal muscle mass in adolescents. On the contrary, they found that a diet rich in saturated fatty acids and sweetened soft drinks was associated with a lower muscle mass, also suggesting an attenuation of the beneficial effects of physical activity on muscle mass accretion during adolescence. Sedentary behaviors are generally accompanied by the consumption of processed foods, which are rich in energy and saturated fats and may negatively influence musculoskeletal health (119). Adolescence is recognized as the period when the bone has the highest responsivity to exercise load, which positively influences skeletal development (120). Physical exercise during bone mass development stimulates bone accretion and appears to delay the onset of osteoporosis during older life. The consequent increase in muscle mass and strength and muscle contraction resulting from physical exercise also determines an increase of bone load and stimulates bone formation (20). Furthermore, longitudinal studies documented that active children had a greater BMD (i.e., +8–10%) in their adulthood compared to the sedentary ones (121). The WHO recommends a minimum of 60 min of moderate to vigorous-intensity physical activity per day in children and adolescents (122). Regarding adult life, it has been suggested that physical activity across adulthood promotes physical performance in midlife and later life. For instance, Cooper et al. (123) reported a cumulative positive effect of physical activity performed in adult life on physical performance later in midlife. Patel et al. (124) found that people with a higher level of physical activity during midlife showed greater physical performance in old age than less active individuals. On the contrary, physically strenuous work in midlife was reported to be a predictor of muscle strength decline after 22 years of follow-up, whereas becoming physically inactive has been associated with an accelerated loss of grip strength (103). Interestingly, the “Dallas Bed Rest and Training” study (125), conducted in 1966 and enrolling 5 healthy 20-year-old male subjects with a 30-year follow-up, found that 3 weeks of bed rest at 20 years of age was detrimental as well as 30 years of aging. In particular, the authors found a significant increase in body fat while fat-free mass did not change. However, they noted that techniques they used at the middle-age assessment may not be able to adequately capture the loss of muscle mass. They also found that the period of bed rest at baseline had a more profound impact on cardiovascular capacity than what was observed at the 30-years of follow-up, with physical inactivity accounting for a greater extent to the decline in aerobic power, although the effect was confounded by the marked increase in body fat. On contrary, the authors demonstrated the beneficial effects of endurance training. This study has immediate clinical implications since it changed clinical practice by minimizing sedentary time when caring for acute and chronic medical conditions (126).

In older adults, it is widely agreed that muscle loss can be counteracted by exercise training. Practice guidelines provide strong recommendations for physical activity as the primary treatment of sarcopenia (127). For older people, structured exercises are recommended to target health-associated physical benefits (5, 23). In particular, resistance training has been proved to reduce insulin resistance and, consequently, promote protein synthesis, increasing muscle mass, strength, and performance (128). Regarding bone health, the most effective type of physical activity is progressive resistance training (weight-lifting and/or resistance bands and cables) and high-impact activity (hopping, skipping, jumping) which help to maintain and increasing BMD in older adults (129). According to the WHO “Global Recommendations on Physical Activity for Health” (122), regular exercise produces benefits both in adults aged 18–64 and in older adults aged 65 and above (with even more benefits in the latter group). Moderate- and vigorous-intensity exercises appear to provide similar health benefits in both groups. Since sarcopenia is defined as a generalized skeletal muscle disorder, it has been recently recommended to perform holistic training involving all muscle groups (130). The WHO recommends to adults aged 65 years and above to perform at least 150 min/week of moderate-intensity physical activity or at least 75 min/week of vigorous-intensity physical activity or an equivalent combination of the two (122). An additional benefit can be obtained by increasing the amount of moderate-intensity physical activity to 300 min/week or to 150 min/week of vigorous-intensity physical activity, and by performing strengthening activities involving the major muscle groups on 2 or more days a week (122). Additionally, an exercise frequency of 2 or more non-consecutive days per week, for at least 3 months has been recommended to significantly improve muscle mass and function. In healthy older adults, an exercise duration of 10 to 15 min per session with eight repetitions for each muscle group has been considered to be sufficient to counteract muscle decline (23, 131). For people with poor mobility, it is suggested to do exercises to enhance balance and prevent falls on 3 or more days per week (122). Exercise training is considered to be safe in older people. However, it has been recommended that it should be supervised in those who are frail or sarcopenic (131). A recent meta-analysis of randomized controlled trials found that long-term exercise training does not influence the risk of dropouts due to health issues or mortality in older adults. On contrary, exercise training results in a reduced mortality risk, decreasing the number of falls and fall-associated injuries and improving physical function (132).

## Conclusion

The age-related musculoskeletal decline (and its adverse consequences) poses an essential burden to individuals and healthcare systems. To date, nutrition and physical exercise remain a mainstay of prevention and intervention for both sarcopenia and osteoporosis, as they are for many other conditions (i.e., ischemic heart disease, diabetes, COPD). Furthermore, there are no formally approved pharmacological agents to prevent or treat sarcopenia. On the other hand, although several drugs exist for the treatment of osteoporosis, they should only be reserved for selected individuals. Implementing preventive strategies even from early life is an emerging area of interest to timely address both the muscle and skeletal decline seen with aging, also associated with early accumulation of fat within muscle mass and the related endocrine-metabolic adaptations. Since early life may represent a window of opportunity in which each person builds up its functional capacities, education toward a healthier lifestyle at a population level may have favorable cascade-like effects, particularly in those living in low socioeconomic conditions. In this period of life, both muscle and bone reach their peak in mass and strength. Maximizing the musculoskeletal peak through adequate nutrition and physical activity at a young age and maintaining the peak in adulthood, is a strategy to counteract the consequent rate of decline seen in older life. Furthermore, preventing the excess of body fat throughout the lifespan is also pivotal, given the negative effects on musculoskeletal health and, more generally, preventing the onset of some chronic conditions later in life. Indeed, implementing a holistic approach to prevention may pave the way to better understand and modify the health trajectories of the individual.

## Author Contributions

DA and GS equally contributed to conceptualizing and writing the manuscript. ES, CL, CA, and MC edited and revised the manuscript. DA, GS, ES, CL, CA, and MC approved the final version of the manuscript. All authors contributed to the article and approved the submitted version.

## Conflict of Interest

MC has received honoraria by Nestlé Health Sciences for presentations at scientific meetings and to serve as a member of Expert Advisory Boards. The remaining authors declare that the research was conducted in the absence of any commercial or financial relationships that could be construed as a potential conflict of interest.

## Publisher's Note

All claims expressed in this article are solely those of the authors and do not necessarily represent those of their affiliated organizations, or those of the publisher, the editors and the reviewers. Any product that may be evaluated in this article, or claim that may be made by its manufacturer, is not guaranteed or endorsed by the publisher.
